# The Story of My Life

**Published:** 1885-02

**Authors:** 


THE STORY OF MY LIFE, by J. Marion Sims, M. D., LL. D. Edited by his son, H. Marion Sims., M. D. D. Appleton & Co., New York: 1884.

In this book we have the life history of the late Dr. Sims up to and including 1863, written by himself in that peculiar pleasant style so conspicuous in the writings of this great man.

Judge Mackey, of Washington, furnishes the introductory and gives in it a resume of this wonderful man's life from 1863 to the day of his death.

We have read the book with interest from the beginning to the end, as every admirer of Dr. Sims will do who is so fortunate as to come into possession of it. It is not alone to the physician that this book will prove interesting and profitable, but to every one who admires the greatness of the "father of American gynecology." We heartily commend the book to our readers.

